# Overuse of coagulation parameter testing in critically ill patients

**DOI:** 10.1186/cc9856

**Published:** 2011-03-11

**Authors:** V Bibeau, F Lauzier, MN Cote, AF Turgeon

**Affiliations:** 1 CHA-Hôpital de l'Enfant-Jésus, Université Laval, Quebec, Canada

## Introduction

The international normalized ratio (INR) is one of the most commonly ordered laboratory tests in the ICU. Recently, it was raised that laboratory tests are widely overused in critically ill patients. We hypothesized that most INRs are inappropriately ordered and could lead to inadequate frozen plasma (FP) transfusion.

## Methods

We performed a retrospective cohort study in a 24-bed medical-surgical ICU of a Canadian teaching hospital. Patients with ≥1 INR testing admitted between 1 January and 30 June 2009 were randomly selected. Admission diagnosis, APACHE II score, drugs affecting coagulation, liver function, invasive procedures, recent or planned surgery, and recent or current bleeding were recorded. INRs ordered for warfarin adjustment were excluded. The primary endpoint was the proportion of inappropriate INRs, based on a blinded assessment of the clinical context by two independent investigators. Secondary endpoints were contributing factors to INR ordering and impact on FP transfusion. We used a standardized case report form. Inter-rater agreement was evaluated using weighted kappa. A third independent investigator resolved disagreement. We used the Student *t *and chi-square tests to compare continuous data and proportions. We obtained ethics approval.

## Results

We included 43 patients (mean age 61.9 ± 16.0, APACHE II score 20.0 ± 8.6, 53.5% males) admitted for nontraumatic bleeding (41.9%), respiratory failure (16.3%), trauma (11.6%), sepsis (11.6%) or other reasons (18.6%). A total of 208 INRs were analyzed, representing 4.9 ± 4.2 INRs per patient. Twenty-five percent of INRs were ordered in the context of bleeding, 6.7% before and 22.1% after surgery, 4.8% before an invasive procedure and 3.8% for suspected liver dysfunction. A total 5.8% of INR were above the normal limit. Inter-rater agreement for INR inappropriateness was good (weighted kappa = 0.61, 95% CI: 0.50 to 0.72). Thirty-one out of 43 (72.1%) patients had at least one INR ordered inappropriately. One hundred and twenty-four out of 208 INRs were inappropriate (59.6%, 95% CI: 52.8 to 66.0). Intravenous heparin was associated with inappropriate INR (RR = 1.47, 95% CI: 1.18 to 1.74). Patients with inappropriate INR had lower APACHE II score (16.9 ± 9.4 vs. 22.8 ± 6.9, *P *= 0.002) and were less likely to receive vasopressors (25.0% vs. 65.2%, *P *= 0.008). No inappropriate INR was followed by FP transfusion.

## Conclusions

Nearly 60% of INR orders were inappropriate. Patients on i.v. heparin, not on vasopressors, and with low APACHE II score were more likely to have inappropriate INRs. Despite no FP transfusion following inappropriate INRs, rationalizing INR testing is warranted to decrease associated costs and resource utilization.

